# Lifestyle and metabolic determinants of early- and late-onset colorectal cancer: insights from a tertiary medical center in Alabama

**DOI:** 10.1007/s10552-026-02213-5

**Published:** 2026-07-16

**Authors:** Pranali G. Patel, Howard W. Wiener, Robert H. Hollis, Carrie G. Lenneman, Sadeep Shrestha

**Affiliations:** 1 Department of Epidemiology, School of Public Health, University of Alabama at Birmingham, Birmingham, AL USA; 2 Department of Surgery, Division of Gastrointestinal Surgery, University of Alabama at Birmingham, Birmingham, AL USA; 3 Division of Cardiology, Department of Medicine, University of Alabama at Birmingham, Birmingham, AL USA

**Keywords:** Early-onset colorectal cancer, Electronic health record, Obesity, Hypertension, Hyperlipidemia, Smoking

## Abstract

**Purpose:**

Early-onset colorectal cancer (EOCRC, < 50 years) is rising in the United States, whereas late-onset CRC (LOCRC, ≥ 50 years) is declining. Fewer studies have examined association between risk factors and age at colorectal (CRC) diagnosis by race. Hence, we investigated whether associations between lifestyle and clinical risk factors and age at CRC diagnosis differed by race.

**Methods:**

Retrospective study conducted between 2010 and 2023 using data from a tertiary medical center in Alabama. Exposure included lifestyle factors—smoking status, and body mass index; clinical factors—type 2 diabetes (T2D), hypertension, and hyperlipidemia. Outcome was age at CRC diagnosis categorized as LOCRC and EOCRC. Multivariable logistic regression was used to examine association between lifestyle and clinical risk factors with age at CRC diagnosis after controlling for race, sex, and marital status. Race-stratified analyses were conducted.

**Results:**

Among 3,209 CRC patients, 54.1% were male, 71.4% White, and 55.8% were married. LOCRC was diagnosed in 81.2%. Patients who were former smokers (OR: 1.89; 95% CI: 1.50, 2.39), had T2D (OR: 1.92; 95% CI: 1.44, 2.57), hypertension (OR: 2.46; 95% CI: 1.65, 3.66), and hyperlipidemia (OR: 3.32; 95% CI: 2.35, 4.70) had higher odds of LOCRC, whereas obesity (OR: 0.61; 95% CI: 0.48, 0.77) had lower odds of LOCRC compared to EOCRC. Hypertension showed significant association with LOCRC in White patients. Hyperlipidemia showed stronger association with LOCRC in Black patients.

**Conclusion:**

Obesity was more prevalent in EOCRC patients and hence these differences should be considered in development of age-stratified screening and prevention strategies.

**Supplementary Information:**

The online version contains supplementary material available at 10.1007/s10552-026-02213-5.

## Introduction

Colorectal cancer (CRC) is the third most common cancer and the second leading cause of cancer-related death in the United States (U.S.) [1, 2]. Several risk factors such as type 2 diabetes (T2D), obesity, hypertension, high red meat consumption, dyslipidemia, smoking, alcohol, and physical inactivity have been associated with an increased risk of CRC [3, 4]. The economic burden of CRC is extensive, with medical care expenditures reaching $23.7 billion and prescription costs totaling $0.6 billion in 2020 alone [5]. These figures reflect the importance of identifying and addressing preventable risk factors to reduce both disease burden and associated healthcare costs.

While CRC primarily affects older adults between 65 and 74 years [1], the incidence of late-onset CRC (LOCRC) referred to as a CRC diagnosis in individuals at age 50 or above, has been declining by approximately 1% per year [6] potentially as a result of improved screening measures and treatment strategies. However, a concerning upward trend of early-onset CRC (EOCRC) cases defined as a CRC diagnosis before age 50 has been observed [7]. From 2018 to 2021, EOCRC incidence increased by 17% compared to 1.2% increase between 2015 and 2018 [8]. This trend signifies a growing public health concern and the need to evaluate and understand factors influencing the increased risk in individuals with EOCRC.

With the increasing incidence of EOCRC, recent studies have further explored whether these factors differ by age at CRC diagnosis across the U.S. For instance, a study from Surveillance, Epidemiology, and End Results (SEER) database between 2000 and 2019 found that diabetes prevalence was an important risk factor for EOCRC whereas physical inactivity was an important risk factor for LOCRC [9]. Findings from ColoCare study found that EOCRC were less likely to be obese (38 vs. 51%) than LOCRC survivors [10]. Moreover, racial disparities persist in CRC incidence across both age groups. EOCRC incidence is disproportionately higher among American Indian/Alaska Native individuals (21%) and non-Hispanic Black individuals (6%) compared to non-Hispanic White individuals [11]. Similarly, LOCRC incidence remains elevated, with rates 29% higher in non-Hispanic Black individuals and 15% higher in AIAN individuals compared to non-Hispanic White individuals [11]. Although these factors are known contributors to overall CRC risk, few studies have examined whether their associations differ between EOCRC and LOCRC, specifically in a high burden region of the U.S.

Importantly, Alabama experiences a disproportionately higher burden of CRC compared to the national average with an age adjusted incidence rate of 40.1 vs. 36.4 per 100,000 [12]. Moreover, Alabama is a part of the “diabetes belt” and “stroke belt,” and is characterized by higher rates of hypertension, T2D, and other risk factors that influence CRC risk [13, 14]. Given Alabama’s elevated CRC burden, it is imperative to focus on these modifiable risk factors. This study aims to investigate lifestyle and clinical-related modifiable risk factors that includes smoking, obesity, T2D, hypertension, and hyperlipidemia between EOCRC and LOCRC patients treated at a tertiary academic medical center in Alabama. In addition, we assessed if these associations varied between Black and White patients. Understanding these associations may provide insights into early detection and prevention strategies for high-risk regions such as Alabama.

## Methods

### Study population

This is a retrospective study in patients diagnosed with CRC between 2010 and 2023 at the tertiary academic medical center in Alabama. The data were received from the University of Alabama at Birmingham (UAB) Informatics for Integrating Biology and the Bedside (i2b2). i2b2 is an NIH-funded National Center for Biomedical Computing based at Partners HealthCare System. It facilitates cohort identification, enabling users to conduct enterprise-wide searches on a de-identified health information repository to find patients who meet specific inclusion or exclusion criteria [15]. Patients with CRC were identified using International Classification of Diseases (ICD) code-10 that included C18.0, C18.2, C18.3, C18.4, C18.5, C18.6, C18.7, C18.8, C18.9, C19.9, and C20.9. The study was approved by UAB Institutional Review Board.

### Exposure

Our exposures of interest were grouped into lifestyle and clinical risk factors. Lifestyle risk factors included smoking status and body mass index (BMI) and clinical risk factors included T2D, hypertension, and hyperlipidemia. Smoking status was categorized as never, former, and current smokers. BMI (kg/m^2^) was categorized using the standard World Health Organization (WHO) classifications as underweight (< 18.5 kg/m^2^), normal weight (18.5–24.9 kg/m^2^), overweight (25.0–29.9 kg/m^2^), and obese (≥ 30.0 kg/m^2^).

Clinical risk factors were identified using ICD 9/10 codes–T2D (250, E10, E11), hypertension (401, I10), and hyperlipidemia (272, E78.5). A binary indicator (yes/no) was assigned for each condition, with presence defined as having the condition recorded prior to the CRC diagnosis. Lifestyle and clinical factors were defined based on documentation recorded prior to the CRC diagnosis. BMI and smoking status reflected the most recent values recorded prior to diagnosis date.

### Outcome

Our dependent variable of interest was age at CRC diagnosis that was categorized as a binary variable: EOCRC was defined as a diagnosis before age 50, while LOCRC was defined as a diagnosis at age 50 or older [16]. LOCRC was selected as the event to facilitate interpretation of associations because most exposure variables were strongly associated with LOCRC within the study cohort.

### Covariates

Covariates of interest included were sex (male and female), self-reported race (Black and White), marital status (single, married, and divorced/separated/widowed) and area deprivation index (ADI). ADI, a validated composite measure of neighborhood-level disadvantage derived from U.S. Census data was used as a proxy for socioeconomic status [17]. The ADI, which incorporates factors such as income, education, employment, and housing quality, was linked to patients’ residential census tract using their geocoded home address. The geocoded addresses were mapped to corresponding census tracts and then merged with national ADI data to assign a deprivation score to each patient. ADI value ranged from 1 (least disadvantaged) to 100 (most disadvantaged) that was categorized as quartiles.

### Statistical analysis

Distribution of the patients’ characteristics overall and stratified by age at CRC diagnosis was reported as count and percentages for categorical variables. Pearson Chi-square test was used to assess the association between covariates and outcome. Multivariable logistic regression was used to examine the association of clinical and lifestyle factors with age at CRC diagnosis (EOCRC and LOCRC). The multivariable model was adjusted for sex, race, and marital status. Odds ratios (OR) and 95% CI were reported.

Stratified analysis was conducted by race to explore potential differences in associations across racial groups, given the well-documented disparities in CRC burden between Black and White patients [11]. In addition, interaction terms between race and each exposure variable were included in the multivariable logistic regression models to formally assess whether associations differed by race. Sensitivity analysis was conducted with ADI added as an additional covariate to assess whether adjustment for socioeconomic status meaningfully influenced the observed associations. ADI was included only in sensitivity analysis due to the high proportion of missing data (~ 39%), which substantially reduced the analytic sample size. Missingness in ADI was largely attributable to unavailable geocoded residential address linkage within the EHR system. We also evaluated differences and trends in patient characteristics across more granular age categories (< 45, 45–49, 50–59, and ≥ 60 years) using Cochran-Armitage trend test and Mantel Haenszel Chi-square test, as appropriate. These age categories were selected to align with current screening guidelines and to reflect clinically relevant distinctions reported in prior literature [18–20]. *p*-values < 0.05 was considered as statistically significant. “Other” races accounted for a small proportion of the population in our cohort (3.52%, *n* = 110) and were excluded to enable appropriate statistical power for comparisons. Only complete observations for each patient were used for the analyses. All statistical analyses were conducted using SAS version 9.4 (SAS Institute Inc., Cary, NC).

## Results

### Cohort characteristics

A total of 3,209 patients with CRC were included in the study. Overall, the majority of the patients were male (54.1%), White (71.4%), and were married (55.8%). Among the total population, 2,604 (81.2%) patients were diagnosed with LOCRC, and 605 (18.8%) patients were diagnosed with EOCRC. Distribution of races was similar in both groups, with White patients comprising 71.0% of LOCRC compared to 73.2% of EOCRC patients (*p* = 0.3058). Patients with LOCRC compared to EOCRC were married (54.4 vs. 61.6%; *p* =  < 0.0001) and resided in ADI Q4 (26.1 vs. 21.4%; *p* = 0.0308). Similar distribution of sex was observed in the two groups (*p* = 0.5804) (Table 1).

**Table 1:** Demographic characteristics of study population

Characteristics	Total (*n* = 3,209)	EOCRC (*n* = 605)	LOCRC (*n* = 2,604)	*p*-value
	*n* (%)	*n* (%)	*n* (%)	
Sex				0.5804
Female	1474 (45.9)	284 (46.9)	1190 (45.7)	
Male	1735 (54.1)	321 (53.1)	1414 (54.3)	
Race				0.3058
Black	861 (28.6)	154 (26.8)	707 (29.0)	
White	2153 (71.4)	420 (73.2)	1733 (71.0)	
Marital status				< 0.0001*
Married	1743 (55.8)	364 (61.6)	1379 (54.4)	
Divorced/widowed/separated	744 (23.8)	54 (9.1)	690 (27.2)	
Single	639 (20.4)	173 (29.3)	466 (18.4)	
ADI				0.0308*
Q1	512 (25.8)	112 (31.5)	400 (24.6)	
Q2	462 (23.3)	75 (21.1)	387 (23.8)	
Q3	508 (25.6)	93 (26.1)	415 (25.5)	
Q4	500 (25.3)	76 (21.4)	424 (26.1)	

ADI quartiles: Q1: ≤ 52, Q2: 53–70, Q3: 71–86, and Q4: ≥ 87

Only complete observations included in the study

*EOCR* early-onset colorectal cancer, *LOCRC* late-onset colorectal cancer, *ADI* area deprivation index, *n* (%) frequency (percentages)

^*^Significance level was set at < 0.05

### Risk factors and differences by age at CRC diagnosis

In adjusted models, being obese was associated with 39% lower odds of LOCRC compared to normal weight (OR: 0.61; 95% CI: 0.48, 0.77; *p* = 0.0009). No significant associations were observed for overweight or underweight categories. Former smoking was associated with 89% higher odds of LOCRC (OR: 1.89; 95% CI: 1.50, 2.39; *p* < 0.0001), while current smoking was non-significant (OR: 1.10; 95% CI: 0.84, 1.43; *p* = 0.0849).

Among clinical factors, hypertension (OR: 2.46; 95% CI: 1.65, 3.66; *p* < 0.0001), T2D (OR: 1.92; 95% CI: 1.44, 2.57; *p* < 0.0001), and hyperlipidemia (OR: 3.32; 95% CI: 2.35, 4.70; *p* < 0.0001) were strongly associated with higher odds of LOCRC compared to EOCRC, respectively (Table 2).

**Table 2:** Logistic regression comparing association between risk factors and age at CRC diagnosis (late-onset, LOCRC vs. early-onset, EOCRC)

Exposures	LOCRC/EOCRC (%)	Adjusted OR^#^ (95% CI)	*p*-value
Lifestyle			
Body mass index			
Underweight	7.1/6.6	0.75 (0.49, 1.14)	0.6542
Overweight	30.8/27.8	0.89 (0.69, 1.15)	0.2154
Obese	31.7/41.2	0.61 (0.48, 0.77)	0.0009*
Normal weight	30.3/24.5	Reference	
Smoking status			
Current	15.8/17.7	1.10 (0.84, 1.43)	0.0849
Former	35.5/22.3	1.89 (1.50, 2.39)	<.0001*
Never	48.7/60.0	Reference	
Clinical			
Hypertension			
Yes	11.9/5.0	2.46 (1.65, 3.66)	<.0001*
No	88.1/95.0	Reference	
Type 2 diabetes			
Yes	19.2/10.4	1.92 (1.44, 2.57)	<.0001*
No	80.8/89.6	Reference	
Hyperlipidemia			
Yes	20.0/6.6	3.32 (2.35, 4.70)	<.0001*
No	80.0/93.4	Reference	

*OR* odds ratio, *CI* confidence intervals, *EOCRC* early-onset colorectal cancer,* LOCRC* late-onset colorectal Cancer, % percentage

^*^Significance level was set at < 0.05

^**#**^Adjusted for sex, race, and marital status

### Risk factors and differences by age at CRC diagnosis among Black and White patients

In race-stratified analyses, several notable differences were observed between Black and White patients (Table 3). Among Black patients, obesity was associated with lower odds of LOCRC (OR: 0.56; 95% CI: 0.34, 0.90), while former smoking (OR: 2.47; 95% CI: 1.51, 4.06), T2D (OR: 1.98; 95% CI: 1.03, 3.82) and hyperlipidemia (OR: 4.83; 95% CI: 2.40, 9.73) were significantly associated with higher odds of LOCRC compared to EOCRC. In contrast, hypertension did not show a statistical significance with age at CRC diagnosis in this group.

**Table 3:** Logistic regression comparing association between risk factors and age at CRC diagnosis (Late-Onset, LOCRC vs Early-Onset, EOCRC) by race

	Black patients (*n* = 861)			White patients (*n* = 2153)			
Exposures	LOCRC/EOCRC (%)	Adjusted OR^#^ (95% CI)	*p*-value	LOCRC/EOCRC (%)	Adjusted OR^#^ (95% CI)	*p*-value	Interaction*p*-value
Lifestyle							
Body mass index							0.9626
Underweight	6.8/8.4	0.62 (0.30, 1.30)	0.5211	5.5/5.2	0.80 (0.48, 1.34)	0.8979	
Overweight	28.1/24.1	0.83 (0.49, 1.40)	0.4605	32.5/30.0	0.90 (0.67, 1.21)	0.3421	
Obese	35.8/47.4	0.56 (0.34, 0.90)	0.0667	31.0/40.0	0.62 (0.47, 0.82)	0.0052*	
Normal weight	29.3/20.1	Reference		31.0/24.8	Reference		
Smoking status							0.3908
Current	16.6/21.2	1.01 (0.62, 1.65)	0.0722	15.3/16.9	1.14 (0.83, 1.57)	0.3446	
Former	32.0/16.5	2.47 (1.51, 4.06)	0.0003*	37.3/25.1	1.75 (1.34, 2.28)	0.0003*	
Never	51.4/62.3	Reference		47.4/58.0	Reference		
Clinical							
Hypertension							0.0955
Yes	18.4/11.0	1.72 (1.00, 2.98)	0.0520	10.0/2.9	3.43 (1.88, 6.24)	0.0004*	
No	81.6/89.0	Reference		90.0/97.1	Reference		
Type 2 diabetes							0.7184
Yes	26.3/14.3	1.98 (1.03, 3.82)	0.0405*	16.8/9.5	2.67 (1.62, 4.40)	0.0001*	
No	73.4/85.7	Reference		83.2/90.5	Reference		
Hyperlipidemia							0.2241
Yes	24.9/5.8	4.83 (2.40, 9.73)	< 0.0001*	18.8/7.1	2.88 (1.93, 4.30)	< 0.0001*	
No	75.1/94.2	Reference		81.2/92.9	Reference		

*OR* odds ratio,* CI* confidence intervals,* EOCRC* early-onset colorectal cancer,* LOCRC* late-onset colorectal cancer, % percentage

^*^Significance level was set at < 0.05 for main effects and interaction effects

^**#**^Adjusted for sex and marital status

Among White patients, similar patterns were observed for obesity (OR: 0.62; 95% CI: 0.47, 0.82), former smoking (OR: 1.75; 95% CI: 1.34, 2.28), T2D (OR: 2.67; 95% CI: 1.62, 4.40), and hyperlipidemia (OR: 2.88; 95% CI: 1.93, 4.30). However, compared to Black patients, hypertension (OR: 3.53; 95% CI: 1.88, 6.24) showed a stronger association with LOCRC in White patients. No significant interactions were observed by race (Table 3).

### Sensitivity analysis of patient characteristics by age at colorectal cancer diagnosis (< 45, 45–49, 50–59, ≥ 60 years)

When examining trends in patients’ characteristics across granular categories of age at CRC diagnosis, no statistically significant trend was observed for sex, race, and smoking status. On the contrary, with increasing age, significant trends were observed for marital status, BMI, hypertension, T2D, and hyperlipidemia. (Supplemental Table 1).

### Sensitivity analyses–risk factors and differences by age at CRC diagnosis adjusting for ADI

After additional adjustment for ADI, results remained largely consistent with the primary model. Obesity was associated with a lower odds of LOCRC (OR: 0.68; 95% CI: 0.50, 0.92), while former smoking was associated with more than two-fold higher odds of LOCRC (OR: 2.09; 95% CI: 1.55, 2.83) compared to EOCRC. Clinical risk factors, including hypertension (OR: 2.80; 95% CI: 1.64, 4.78), T2D (OR: 2.33; 95% CI: 1.57, 3.46), and hyperlipidemia (OR: 2.34; 95% CI: 1.53, 3.56), were also significantly associated with LOCRC compared to EOCRC (Supplemental Table 2).

## Discussion

The study investigated the association of lifestyle and clinical risk factors with age at CRC diagnosis in a diverse population of a tertiary academic medical center in Alabama. We found that patients with LOCRC were more likely to have a normal BMI, be former smokers, have a history of T2D, hypertension, and hyperlipidemia compared to EOCRC. These differences remained persistent across stratified and sensitivity analyses underscoring the interplay between lifestyle and clinical factors in shaping age-related differences in CRC presentation. Notably, after adjusting for ADI, a proxy for socioeconomic status, the effect estimates did not show any meaningful difference suggesting that ADI did not account for the observed associations.

Our findings align and extend prior work on age-related differences in CRC risk factors by examining differences in risk profiles between LOCRC and EOCRC patients thereby highlighting variations in clinical presentations. Results from the ColoCare cohort study reported that EOCRC survivors were less likely to be ever smokers compared to (38 vs. 51%, *p* < 0.001) LOCRC [10] that was consistent with our study findings that indicated LOCRC had increased odds of being former smokers compared to never smokers. This association was consistent in both Black and White patients in our study, though the effect was stronger among Black patients, suggesting possible differences in smoking exposure or cessation patterns by race.

Contrary to our findings that reported higher odds of obesity in the EOCRC patients, the ColoCare study reported lower rates of obesity (27 vs. 31%, *p* < 0.001) among EOCRC compared to LOCRC patients [10]. In addition, a 2021 systematic review and meta-analysis found that overweight and obese compared to average weight younger adults had 32 and 88% higher risk of developing CRC, respectively [21]. Another study from Kaiser Permanente Southern California reported that obesity was significantly associated with increased risk of early-onset colorectal adenocarcinoma compared to non-cancer controls [22]. In our race-stratified analyses, obesity was strongly associated with EOCRC than LOCRC in both Black and White patients, consistent with our overall findings and suggesting that excess body weight may be more strongly linked to EOCRC. These inconsistent findings regarding obesity highlight the need for further investigation to better understand its role in CRC risk by age of diagnosis, specifically as regional variations such as those observed in Alabama [23] may explain the differences compared to national cohorts.

With respect to clinical factors, our results were consistent with findings from Taiwan’s national cancer registry study that reported higher prevalence of hypertension (35.6 vs. 8.2%) and T2D (17.9 vs. 4.5%) among LOCRC compared to EOCRC patients [24]. Similarly, in the study from two medical centers (Hospital Alma Máter de Antioquia and Clínica Medellín) in Medellín, Colombia, the authors reported higher prevalence of arterial hypertension (49.1 vs. 6.7%) and T2D (22.1 vs. 6.7%) in the LOCRC compared to EOCRC patients [25]. These global findings are consistent with our study results reflecting the stronger association between LOCRC with hypertension and T2D. When stratified by race, however, hypertension was strongly associated with LOCRC among White patients but not Black patients. This difference may reflect earlier onset of hypertension among Black adults [26–28] and poorer BP control despite similar awareness and treatment rates [29, 30], which could reduce its utility in distinguishing LOCRC from EOCRC in this group. Furthermore, a study from SEER between 2000 and 2019 reported that diabetes prevalence was the most important risk factor in predicting EOCRC compared to LOCRC, which was inconsistent with our study results. One possible explanation for these differences between EOCRC and LOCRC could be due to differences in study populations, and data sources than the underlying biological mechanism. In our study, T2D was significantly associated with LOCRC in both racial groups with comparable effect sizes, further reinforcing the role of metabolic dysregulation in age-related CRC risk.

Further, a meta-analysis of 24 studies found that higher vs. lower levels of serum total cholesterol [relative risk ((RR): 1.15; 95% CI 1.08, 1.22)] and triglycerides (RR: 1.21; 95% CI 1.09, 1.34) were associated with overall CRC risk [31]. This aligns with our findings that reported hyperlipidemia to be strongly associated with LOCRC, consistent with the fact CRC occurs more often in older adults and lipid abnormalities become more prevalent with advancing age [32]. In our cohort, hyperlipidemia was strongly associated with LOCRC in both Black and White patients, though the effect appeared stronger among Black patients, consistent with racial differences in lipid levels [33]. This abnormal lipid metabolism can serve as a clinical marker of distinct metabolic risk profiles across age groups. Our study is among the first to examine hyperlipidemia in relation to age at CRC diagnosis, evaluating its potential as a distinguishing factor.

Although EOCRC patients in our cohort were less likely to have T2D, hypertension, and hyperlipidemia, it does not imply a lower risk profile. For instance, national estimates indicate that approximately 4% of adults younger than 50 years have diabetes (defined as hemoglobin A1C of 6.5% or greater or a healthcare diagnosis of diabetes) [34], whereas the prevalence of T2D among EOCRC patients in our cohort was 10.4%, indicating a higher burden than expected for this age group. This finding suggests that metabolic dysregulation may contribute to EOCRC risk, even when associations appear stronger among LOCRC patients. At the same time, EOCRC may be driven by alternate mechanisms. Prior research has reported that individuals with EOCRC may have distinct profiles than LOCRC and are found to be linked to genetic predispositions (e.g., Lynch syndrome, familial adenomatous polyposis), early-life exposures, or microbiome-related factors [35, 36]. These differences in EOCRC and LOCRC necessitate age-specific screening strategies as current guidelines recommend screening initiation at age 45. Therefore, it is important to identify factors influencing increased burden of CRC across age groups to provide tailored preventive and interventional measures.

The study has a few limitations. First, the use of EHR data may introduce misclassification bias due to use of ICD codes for disease identification. Second, due to the cross-sectional study design, we could not infer causality. Third, we did not consider other factors such as diet, physical activity, or environmental exposures as we did not have detailed information to measure the association. Fourth, the study population was derived from a tertiary academic medical center in Alabama and therefore may differ from the broader Alabama population in referral patterns, disease severity, or access to specialty care, which may limit generalizability. In addition, Alabama has a higher baseline prevalence of metabolic comorbidities such as hypertension, T2D, and hyperlipidemia compared to many other regions of the U.S., which may influence the observed associations. Lastly, since this study compared LOCRC and EOCRC within a CRC cohort, the clinical factors evaluated may partially reflect age-related comorbidity burden rather than independent determinants of age at diagnosis. Despite these limitations, the study has a few strengths. This study utilizes a diverse patient population from one of the largest tertiary academic medical center in the southeastern U.S., a region with a disproportionately high burden of CRC, which enhances the clinical relevance of our findings. Despite the chances of misclassification bias, the use of ICD codes enabled easier identification of diseases and ensured consistency in disease classification thereby enhancing reproducibility in similar healthcare studies. Furthermore, our study contributes novel insights by assessing hyperlipidemia as a potential risk factor in relation to the age at CRC diagnosis.

## Conclusion

Our study provides strong public health implications by explaining the differences between LOCRC and EOCRC by risk factors in a population with a higher burden of CRC. These study findings are particularly critical for Alabama, a state located in the “Diabetes belt” and “Stroke belt” with existing health disparities and barriers to preventive services. With the increasing incidence of EOCRC and distinct risk profile, future studies should explore gene-environment interactions and the role of early-life exposures to better understand the underlying etiology.

## Supplementary Information

Below is the link to the electronic supplementary material.

Supplementary file1 (DOCX 21 kb)

## Data Availability

The authors do not have permission to share data.
