# Supplementary material for: Lifestyle and metabolic determinants of early- and late-onset colorectal cancer: insights from a tertiary medical center in Alabama
Source: Cancer Causes Control. 2026 Jul 16;37(8):127. doi: 10.1007/s10552-026-02213-5 (PMC13375673; doi:10.1007/s10552-026-02213-5)
Supplement: Supplementary file 1 — Supplementary file1 (DOCX 21 kb) [file 10552_2026_2213_MOESM1_ESM.docx]

**Lifestyle and Metabolic Determinants of Early- and Late-Onset Colorectal Cancer**

Pranali G. Patel^1^, Howard. H. Wiener^1^, Robert Hollis^2^, Carrie G. Lenneman^3^, Sadeep Shrestha^1^

**Table of Contents**

Supplemental Table 1. Patient Characteristics Across Colorectal Cancer Diagnosis Age (<45, 45-49, 50-59, ≥60 Years)

Supplemental Table 2. Logistic Regression comparing association between Risk Factors and Age at CRC Diagnosis (Late Onset, LOCRC vs Early Onset, EOCRC)

|  | **Age at Colorectal Cancer Diagnosis** | | | | |
| --- | --- | --- | --- | --- | --- |
| **Characteristics** | **<45 years** | **45-49 years** | **50-59 years** | **≥60 years** |  |
|  | **N (%)** | **N (%)** | **N (%)** | **N (%)** | ***p*-value** |
| ***Demographic*** | | | | |  |
| Sex |  |  |  |  | 0.7639 |
| Female | 160 (46.5) | 124 (47.5) | 409 (45.3) | 781 (45.9) |  |
| Male | 184 (53.5) | 137 (52.5) | 494 (54.7) | 920 (54.1) |  |
| Race |  |  |  |  | 0.6695 |
| Black | 86 (26.5) | 68 (27.3) | 277 (33.0) | 430 (26.9) |  |
| White | 239 (73.5) | 181 (72.7) | 562 (67.0) | 1171 (73.1) |  |
| Marital Status |  |  |  |  | 0.0035^*^ |
| Married | 201 (59.6) | 163 (64.2) | 484 (54.9) | 895 (54.1) |  |
| Divorced/Widowed/Separated | 25 (7.4) | 29 (11.4) | 159 (18.1) | 531 (32.1) |  |
| Single | 111 (32.9) | 62 (24.4) | 238 (27.0) | 228 (13.8) |  |
| ***Lifestyle*** | | | | |  |
| Body Mass Index |  |  |  |  | <0.0001^*^ |
| Underweight | 23 (6.7) | 17 (6.5) | 70 (7.8) | 116 (6.8) |  |
| Normal weight | 81 (23.6) | 67 (25.7) | 224 (24.8) | 565 (33.2) |  |
| Overweight | 96 (27.9) | 72 (27.6) | 269 (29.8) | 533 (31.3) |  |
| Obese | 144 (41.9) | 105 (40.2) | 340 (37.7) | 487 (28.6) |  |
| Smoking status |  |  |  |  | 0.2790 |
| Current | 58 (17.6) | 45 (17.9) | 205 (24.0) | 186 (11.5) |  |
| Former | 75 (22.7) | 55 (21.8) | 237 (27.7) | 642 (39.7) |  |
| Never | 197 (59.7) | 152 (60.3) | 413 (48.3) | 791 (48.9) |  |
| ***Clinical*** | | | | |  |
| Hypertension |  |  |  |  | <0.0001^*^ |
| Yes | 12 (3.5) | 18 (6.9) | 76 (8.4) | 235 (13.8) |  |
| Type 2 diabetes |  |  |  |  | <0.0001^*^ |
| Yes | 31 (9.0) | 32 (12.3) | 154 (17.1) | 345 (20.3) |  |
| Hyperlipidemia |  |  |  |  | <0.0001^*^ |
| Yes | 17 (4.9) | 23 (8.8) | 134 (14.8) | 386 (22.7) |  |
| *N (%) - Frequency (Percentages)*  **p<0.05 as test of significance* | | | | | |

| **Exposures** | **Adjusted OR^#^ (95% CI)** | **p-value** |
| --- | --- | --- |
| ***Lifestyle*** | | |
| Body Mass Index |  |  |
| Underweight | 0.86 (0.50, 1.47) | 0.9932 |
| Overweight | 0.93 (0.67, 1.29) | 0.4613 |
| Obese | 0.68 (0.50, 0.92) | 0.0268 |
| Normal weight | Reference |  |
| Smoking status |  |  |
| Current | 1.23 (0.86, 1.76) | 0.3582 |
| Former | 2.09 (1.55, 2.83) | <0.0001 |
| Never | Reference |  |
| ***Clinical*** | | |
| Hypertension |  |  |
| Yes | 2.80 (1.64, 4.78) | 0.0002 |
| No | Reference |  |
| Type 2 diabetes |  |  |
| Yes | 2.33 (1.57, 3.46) | <0.0001 |
| No | Reference |  |
| Hyperlipidemia |  |  |
| Yes | 2.34 (1.53, 3.56) | <0.0001 |
| No | Reference |  |
| *Abbreviations: OR, Odds Ratio; CI, Confidence Intervals*  **^#^***Adjusted for sex, race, marital status, and ADI* | | |
